# The complete genome sequence of *Toxicodendron radicans*, Eastern Poison Ivy

**DOI:** 10.12688/f1000research.25556.1

**Published:** 2020-08-20

**Authors:** Toby Pirro, Stacy Pirro

**Affiliations:** 1 Biodiversity, IRIDIAN GENOMES, Bethesda, MD, 20817, USA

**Keywords:** Toxicodendron radicans, Eastern Poison Ivy, genome, assembly, annotation

## Abstract

Eastern Poison Ivy ( *Toxicodendron radicans*, Anacardiaceae) is well known in Eastern North America for causing contact dermatitis, an itchy and painful rash in most people who come in contact with it.  We present the whole genome sequence and annotation of this species. A total of 96,255,779 paired-ends reads consisting of 28.9 G bases were obtained by sequencing one leaf from a wild-collected plant.  The reads were assembled by a *de novo* method followed by alignment to related species. Annotation was performed via GenMark-ES. The raw and assembled data is publicly available via GenBank: Sequence Read Archive ( SRR10325927) and Assembly ( GCA_009867345).

## Introduction

Eastern Poison Ivy ( *Toxicodendron radicans*, Anacardiaceae) is well known in the Eastern United States, Canada, Mexico and parts of China for causing contact dermatitis, an itchy and painful rash in most of the human population ( Barceloux, 2008). The rash, along with accompanying blisters, is caused by urushiol, an oil compound in the plant's sap. Urushiol-induced allergic rashes are a Type IV hypersensitivity reaction ( Kalish & Johnson, 1990). This type of reaction is a cell-mediated response and can take hours to days to produce symptoms ( Williams *et al.*, 1999). Approximately 15% of people have no allergic reaction to urushiol, but most people experience a reaction from between 5–12 days after exposure. Allergic reactions can increase in duration and severity after each incident ( Bonnekoh *et al.*, 2019).

Exposure to the urushiol in *Toxicodendron radicans* can cause hypersensitivity in related plants, such as mango, *Mangifera indica* ( Yoo & Carius, 2019), and the Chinese Lacquer Tree, *Toxicodendron vernicifluum*. Consumer products made from the Chinese Lacquer Tree are manufactured by curing the urushiol-containing sap to a clear, hard, waterproof substance. Improperly cured products can cause contact dermatitis in urushiol-hypersensitive people several years after manufacture, and present an ongoing health challenge to lacquerware workers ( Ma *et al.*, 2012).

A complete genome sequence for this species will allow the insight into the evolution of the urushiol biosynthetic pathway.

## Methods

A leaf from a single wild-collected *Toxicodendron radicans* plant was used as the source of genomic DNA. Extraction was performed on tissue from a single leaf using the Qiagen DNAeasy genomic extraction kit for plants, using the standard process. A paired-end sequencing library was constructed using the Illumina TruSeq kit, according to the manufacturer’s instructions. The library was sequenced on an Illumina Hi-Seq platform in paired-end, 2 × 150bp format. 

The resulting fastq files were trimmed of adapter/primer sequence and low-quality regions with Trimmomatic (v0.33) ( Bolger *et al.*, 2014). The trimmed sequence was assembled by SPAdes (v2.5) ( Bankevich *et al.*, 2012) followed by a finishing step using RagTag (v1.0.0) ( Alonge, 2020) to make additional contig joins based on conserved regions in related plant species: *Mangifera indica* (mango, GCA_011075055) and *Pistacia vera* (pistachio, GCA_008641045). Default procedures were used for all assembly steps.

Annotation was performed using GeneMark-ES (v2.0) ( Lomsadze *et al.*, 2005). Annotation was performed fully *de novo* without a curated training set and default parameters.

## Results

The genome assembly yielded a total sequence length of 454,874,194 bp over 270,263 scaffolds with an N50 of 1,945,245. The GeneMark-ES annotation resulted in 42,021 genes.

## Data availability

### Underlying data

Raw and assembled data is publicly available via GenBank:

Raw genome of *Toxicodendron radican*, Accession number SRR10325927: https://www.ncbi.nlm.nih.gov/sra/?term=SRR10325927

Assembly of *Toxicodendron radican*, Accession number GCA_009867345: https://www.ncbi.nlm.nih.gov/assembly/GCA_009867345.1/
