# Supplementary material for: The complete genome sequence of Toxicodendron radicans, Eastern Poison Ivy
Source: F1000Res. 2020 Aug 20;9:1015. [Version 1] doi: 10.12688/f1000research.25556.1 (PMC8226398; doi:10.12688/f1000research.25556.1)
Supplement: Peer review history

# Peer review: Reviewer response for version 1

**DOI:** 10.5256/f1000research.28204.r87603

**Published:** 2021-07-07

**Authors:** Hyungtaek Jung

**Affiliations:** 1 School of Biological Sciences, The University of Queensland, St Lucia, Qld, Australia

The authors have assembled the genome by employing Illumina sequencing technology using the leaf tissue of *T*. *radicans*. There is no doubt that the data generated in the current study will make a valuable contribution to the scientific community, however, I have several concerns about the analyses and/or the interpretation of the data as it currently stands.

**Methods:**

 The fact is Illumina-based genome assemblies contain many misassemblies. Given the circumstances, why did authors choose Illumina reads over PacBio and Oxford Nanopore? What’s the overall sequencing and assembly summary? Any chloroplast DNA contamination? If yes, how did you treat them? Where are the executed scripts? Annotation with GeneMark-ES without any RNA-Seqs?

**Results:**

 How did you evaluate the assembly accuracy (e.g. BUSCO)? Where are the structural (RepeatMasker) and functional annotations? It needs more explanation.

Are sufficient details of methods and materials provided to allow replication by others?

Partly

Is the rationale for creating the dataset(s) clearly described?

Partly

Are the datasets clearly presented in a useable and accessible format?

Yes

Are the protocols appropriate and is the work technically sound?

Partly

Reviewer Expertise:

Genomics and Bioinformatics

I confirm that I have read this submission and believe that I have an appropriate level of expertise to confirm that it is of an acceptable scientific standard, however I have significant reservations, as outlined above.

# Peer review: Reviewer response for version 1

**DOI:** 10.5256/f1000research.28204.r87604

**Published:** 2021-06-24

**Authors:** Xuming Zhou

**Affiliations:** 1 Key Laboratory of Animal Ecology and Conservation Biology, Chinese Academy of Sciences, Beijing, China

This study reported the complete genome sequence of *Toxicodendron radicans*, which could help future research of this species. The manuscript is well written and should be considered for indexing prior to two questions:

1. The author should provide more details about the annotation, for example, how *de novo* and homologous approaches were performed.
2. The author should compare their genome quality to their relative species.

Are sufficient details of methods and materials provided to allow replication by others?

No

Is the rationale for creating the dataset(s) clearly described?

Yes

Are the datasets clearly presented in a useable and accessible format?

Yes

Are the protocols appropriate and is the work technically sound?

Yes

Reviewer Expertise:

Genomics, Phylogenetics, Aging

I confirm that I have read this submission and believe that I have an appropriate level of expertise to confirm that it is of an acceptable scientific standard.

# Peer review: Reviewer response for version 1

**DOI:** 10.5256/f1000research.28204.r73876

**Published:** 2020-11-13

**Authors:** Rongxi Sun

**Affiliations:** 1 Jiangxi Agricultural University, Nanchang, China

I have now thoroughly reviewed the manuscript “The complete genome sequence of *Toxicodendron radicans*, Eastern Poison Ivy”. Given urushiol in this plant’ sap causing dermatitis, an itchy and painful rash in most of the human population, the new assembly of complete genome sequence of this species helps to understand the evolution of the urushiol biosynthetic pathway. However, there are still some flaws in the manuscripts which the authors may consider to change:

1. Method: What is the source of the leaf used for sequencing, such as tree age and specific location.
2. Results：How accurate is this high-throughput genome sequence? Whether there are indicators related to accuracy in the results.
3. In results, the annotation should be more discussed.

Are sufficient details of methods and materials provided to allow replication by others?

Yes

Is the rationale for creating the dataset(s) clearly described?

Partly

Are the datasets clearly presented in a useable and accessible format?

Yes

Are the protocols appropriate and is the work technically sound?

Partly

Reviewer Expertise:

Genetic diversity and phylogeography

I confirm that I have read this submission and believe that I have an appropriate level of expertise to confirm that it is of an acceptable scientific standard.
